# Impact of the COVID-19 Pandemic on the Detection of Leprosy in Micro-Regions with a High Risk of Illness in Minas Gerais, Brazil

**DOI:** 10.3390/idr16060089

**Published:** 2024-11-26

**Authors:** Sarah Lamas Vidal, Daniele dos Santos Lages, Isabela Cristina Lana Maciel, Isabel Cristina Gonçalves Leite, Angélica da Conceição Oliveira Coelho, Francisco Carlos Félix Lana

**Affiliations:** 1 Postgraduate Program in Nursing, School of Nursing, Federal University of Minas Gerais, Belo Horizonte 30130-100, Minas Gerais, Brazil; sarahlamasvidal@ufmg.br (S.L.V.); daniele-lages@ufmg.br (D.d.S.L.); isabelaclm@ufmg.br (I.C.L.M.); 2 Department of Public Health, School of Medicine, Federal University of Juiz de Fora, Juiz de Fora 36036-900, Minas Gerais, Brazil; isabel.leite@ufjf.br; 3 Department of Basic Nursing, Faculty of Nursing, Federal University of Juiz de Fora, Juiz de Fora 36036-900, Minas Gerais, Brazil; angelica.coelho@ufjf.br; 4 Department of Maternal and Child Nursing and Public Health, School of Nursing, Federal University of Minas Gerais, Belo Horizonte 30130-100, Minas Gerais, Brazil

**Keywords:** leprosy, epidemiology, COVID-19 pandemic, public health surveillance

## Abstract

Background: Leprosy control remains challenging in Brazil and has been aggravated by the COVID-19 pandemic. Objective: To analyze the impact of the COVID-19 pandemic on the epidemiological scenario of leprosy through the detection rate of new cases, the risk of illness, and the hidden prevalence of leprosy according to high-risk micro-region in Minas Gerais, Brazil. Methods: An ecological study conducted in the health micro-regions of Minas Gerais, using data on new leprosy cases diagnosed between 2015 and 2023. The annual detection rate of new cases, the risk of illness index and the hidden prevalence of leprosy were evaluated. The time trend was evaluated by calculating the annual percentage change (APC) of the detection rate, based on segmented linear regression, considered significant when it showed *p* < 0.05. Results: The state of Minas Gerais showed a significant negative annual increase between 2015 and 2020 (APC = −7.91; 95%CI −21.76–−1.72), and in 2020 it showed an inflection point, with an annual increase of 9.91 in the period from 2020 to 2023. When evaluating the hidden prevalence of leprosy in Minas Gerais, we observed a reduction in the estimates’ average from 2015–2019 (2.78) to 2020–2023 (2.00). The state as a whole showed an upward trend in the risk of illness, with the average index varying from 0.28 (medium risk) to 0.55 (high risk). Conclusions: The pandemic has had a considerable and heterogeneous impact on the detection of new cases, as well as on the risk of becoming ill and the hidden prevalence of leprosy, with repercussions for the control of the endemic in populations. There is a need to implement public health policies that prioritize the early identification of cases and ensure that vulnerable populations are monitored.

## 1. Introduction

Leprosy is a disease which has genetic determinants [1], and immunological [2] and socioeconomic [3,4] factors linked to the risk of becoming ill. It is historically neglected and still endemic in many countries. A drop in detection of new cases was intensified between 2019 and 2021 [5]. With the COVID-19 pandemic, health services had to reorganize. Isolation measures, as well as the suspension of active searches by services, had a strong impact on the early detection of leprosy cases [6]. So, strengthening leprosy surveillance and control actions is of great importance in the early detection of new cases, which contributes to reducing disabilities and the stigma related to the disease [7].

In Brazil, the COVID-19 pandemic advanced exponentially [8]. Some diseases, including leprosy, have had their care adapted with the provision of treatment extended by two or three months, consultations, and remote care, in order to guarantee the home isolation of these people [9]. In addition, reference hospitals for leprosy changed their care flows during the critical period of the pandemic, allocating 100% of their beds to care for patients affected by or suspected of COVID-19 [10].

As a result of the impacts of the pandemic, in Brazil there was a reduction in the diagnosis of leprosy, as well as an increase in cases in the multibacillary clinical form, which is mainly responsible for maintaining the chain of transmission, demonstrating that control strategies had a strong impact in the country [11]. There was also a reduction in the assessment of the degree of disability among diagnosed cases [12], which indicates significant operational weaknesses in the surveillance of disabilities.

Despite the reduction in the detection rate caused by the impact of the pandemic, Brazil is still among the three countries that diagnose the most leprosy cases in the world. In 2022, 19,635 new cases of the disease were diagnosed, of which 836 were in children under 15 and 1917 had grade 2 disabilities at the time of diagnosis [5].

In order to overcome this scenario, and in line with the WHO Global Strategy for Leprosy 2021–2030 called “Towards zero leprosy” [13], the National Strategy for Tackling Leprosy 2024–2030 was drawn up in Brazil, with the vision of “a Brazil without leprosy” [14]. In Minas Gerais, the Minas Gerais State Department of Health (SES-MG) proposed the “2019–2022 State Plan for Tackling Leprosy” before the pandemic, which established strategies for tackling leprosy according to the needs of each point in the care network in order to outline a horizontal and hierarchical network [15].

Despite social determination, the “risk of becoming ill” from leprosy is strongly impacted by the operational capacity of health services to carry out programmatic health actions. This concept involves epidemiological factors that reflect the magnitude and strength of the morbidity of the endemic disease, as well as factors related to the operational capacity of health services to carry out disease control actions [16]. In addition to the risk of becoming ill, the hidden prevalence of leprosy also reflects the limitations of the health services in carrying out leprosy control actions and timely diagnosis, since the estimate is obtained from data on cases diagnosed with disabilities, which represent late diagnosis and, in turn, suggest the presence of other undetected cases [17].

The quality of leprosy control actions has an effect on disease monitoring indicators [7]. Based on the weaknesses exposed above, the aim of this study was to analyze the impact of the COVID-19 pandemic on the epidemiology of leprosy through the detection rate of new cases, the risk of illness, and the hidden prevalence of leprosy according to high-risk micro-region in Minas Gerais, Brazil.

## 2. Materials and Methods

### 2.1. Study Settings and Period

This is an ecological study conducted in the health micro-regions of Minas Gerais, Brazil. The focus of the study is the state of Minas Gerais and its micro-regions classified as being at “High Risk” of becoming ill with leprosy, according to the analysis of data on notifications of new cases of the disease diagnosed between 2015 and 2023, and according to the 89 micro-regions. The micro-regions were classified into different levels of risk, with “High Risk” defined as those located in the upper quartile of the index distribution.

### 2.2. Study Participants

The study population consisted of new leprosy cases notified on the Notifiable Diseases Information System (SINAN) in Minas Gerais. Records of international, inter-municipal, and inter-state transfers, readmissions, and diagnostic errors were excluded.

### 2.3. Data Collection Process

Secondary data on the notification of new leprosy cases were taken from the SUS Information Technology Department (DATASUS)—TABWIN, Brazil. Demographic data relating to the general population, necessary for obtaining epidemiological indicators for leprosy in the micro-regions, were extracted from the IBGE database, via the DATASUS website, from the 2010 demographic census and intercensal projections. These extracted secondary data were entered into a database created using Microsoft Excel software (version 2010) to construct the study variables.

### 2.4. Data Analysis

To assess the impact of the COVID-19 pandemic on the epidemiological scenario of leprosy, the following indicators were evaluated: annual detection rate of new cases, per 100,000 inhabitants, which makes it possible to measure the strength of morbidity, magnitude, and trend of the disease [18]; the Leprosy Risk Index [16], which integrates three epidemiological indicators, making it possible, in addition to the evaluation based on the detection rate of new cases, to also evaluate the strength of recent transmission and the timely detection of new leprosy cases [18]; and the hidden prevalence of leprosy [19], which represents the reservoir of undetected cases [17].

In order to assess temporal trends in the period, the annual percentage change (APC) of the annual detection rate of new leprosy cases between 2015 and 2023 was calculated. The annual percent change (APC) estimated from the coefficients is a summary of trends in rates over short time intervals, estimated from segmented linear regression (joinpoint regression), using the Joinpoint Regression statistical program, version 5.0.2 (Statistical Research and Applications Branch, National Cancer Institute, Rockville, MD, USA). This regression model makes it possible to estimate the average annual variation for the study period and the points (years) at which the trend changes. The program uses minimum and maximum junction points (it starts with the zero point) and tests with other junction points (until it reaches the maximum number), checking whether the changes are statistically significant. The significance test uses the Monte Carlo permutation method. This permutation tests a set of data to select junction points. This makes it possible to verify the existence of inflection points in the trend of this rate, considering the period 2015–2019 (pre-pandemic) and 2020–2023 (pandemic phase).

The estimate of the hidden prevalence of leprosy, according to the methodology proposed by Suárez and Lombardi (1997), is based on the assumption that the detection of cases with disabilities indicates late detection and therefore cases that should have been detected.

The Disease Risk Index is a composite indicator that integrates three of the main leprosy monitoring indicators: (i) annual detection rate of new leprosy cases, (ii) annual detection rate of new leprosy cases in the population aged 0–14, and (iii) rate of new leprosy cases with grade 2 disability at the time of diagnosis [20].

To analyze the hidden prevalence of leprosy and the risk of leprosy disease, the study period was divided into two segments: (1) 2015–2019, representing the pre-pandemic phase, and (2) 2020–2023, representing the pandemic phase. From this perspective, the study variables were used considering segments 1 and 2, based on the average annual detection for each period.

### 2.5. Ethical Consideration

The data used in this study were secondary data obtained from public portals of the federal government, so it was not necessary to use an informed consent form.

## 3. Results

The results of the analysis of the impact of the COVID-19 pandemic on the epidemiology of leprosy will be presented through the detection rate of new cases, the risk of illness, and the hidden prevalence of leprosy according to high-risk micro-region in Minas Gerais, Brazil. There was a reduction in the average detection of new cases in the state rate. This same variation was observed among the micro-regions, with the exception of São Gotardo. These data are shown in Table 1.

Analyzing the annual increase (APC) in the overall detection rate over the entire period (2015 to 2023), seven of these micro-regions showed a significant downward trend (*p* < 0.05): Almenara/Jacinto (APC = −9.25; 95%CI −16.61–−1.10), Araçuaí (APC = −11.79; 95%CI −17.85–−5.22), Belo Horizonte/Nova Lima/Caeté (APC = −9.61; 95%CI −14.65–−4.16), Carangola (APC = −12.83; 95%CI −23.67–−0.26), Ipatinga (APC = −9.32; 95%CI −14.74–−3.43), Uberlândia/Araguari (APC = −8.98; 95%CI −17.02–0.00) and Unaí (APC = −6.95; 95%CI −12.03–−1.42).

**Figure 1:**
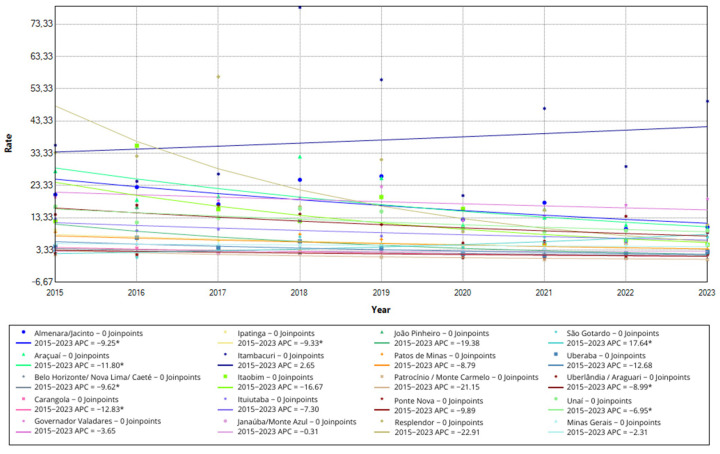
Overall detection rate of new leprosy cases in in Minas Gerais and health micro-regions, multiple joinpoint models, 2015 to 2023. Legend: The dots on the graph indicate the value of the overall detection rate of new leprosy cases in each year. The lines represent the trend of the rate over the study period in each of the micro-regions. Below the graph is the color that represents each of the micro-regions, as well as the value of the annual increase over the study period. The * represents significative change in the annual increase.

On the other hand, the São Gotardo micro-region showed a significant growth trend throughout the period, with an annual increase of 17.64 (95%CI 2.75–34.90). These results reinforce the variation in local transmission dynamics, with most regions showing a significant reduction, while São Gotardo went in the opposite direction. The trend in the overall detection rate in the nineteen micro-regions, as well as the state rate, is shown in Figure 1.

When evaluating the inflection points observed over the period, we identified a trend towards recovery. The state of Minas Gerais showed a significant negative annual increase between 2015 and 2020 (APC = −7.91; 95%CI −21.76–−1.72), and in 2020 it showed an inflection point, with an annual increase of 9.91 in the period from 2020 to 2023.

**Figure 2:**
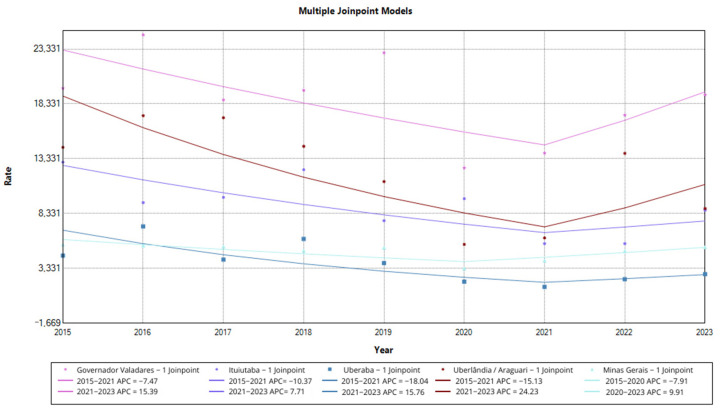
Recovery trend in the overall detection rate of new leprosy cases in Minas Gerais and health micro-regions, multiple joinpoint models, 2015 to 2023. Legend: The dots on the graph indicate the value of the overall detection rate of new leprosy cases in each year. The lines represent the trend of the rate over the study period in each of the micro-regions. Below the graph is the color that represents each of the micro-regions highlighted in the model. It also shows the value of the annual increase, highlighting the moment in the historical series when the rate reached a turning point. The * represents significative change in the annual increase.

The micro-regions of Governador Valadares, Ituiutaba, Uberaba, and Uberlândia/Araguari, which showed a negative increase between 2015 and 2021, showed a positive annual increase in their detection rate between 2021 and 2023. These changes were not statistically significant, but they suggest a trend towards a resumption in the diagnosis of new leprosy cases in these regions. This result is illustrated in Figure 2.

Analysis of the risk of falling ill with leprosy between the 2015–2019 and 2020–2023 periods revealed significant changes in the classification of this indicator in nine health micro-regions in Minas Gerais. The micro-regions of Carangola, Itaobim, Ituiutaba, Patos de Minas, and Resplendor, which previously had a high risk of falling ill, became medium-risk during the pandemic period.

Ponte Nova and Uberaba went from a high to low risk of illness, with Uberaba registering a decrease from 0.51 to 0.35. The micro-regions of João Pinheiro and Patrocínio/Monte Carmelo stood out for their even sharper reduction, going from a high to a very low risk of illness, with indices of 0.46 and 0.54, falling to 0.11 and 0.17, respectively. The changes in the index reflect a significant change in the pattern of illness.

While these areas showed a drop in the risk of illness, the other micro-regions evaluated maintained their high-risk classification, with no significant changes between the two periods analyzed. The state as a whole showed an upward trend in the risk of illness, with the average index varying from 0.28 (medium risk) to 0.55 (high risk), showing a worrying scenario in some regions (Table 2).

When evaluating the hidden prevalence of leprosy in the micro-regions of Minas Gerais during the period from 2015 to 2023, we observed a reduction in the estimates both statewide and for several micro-regions, including Belo Horizonte/Nova Lima/Caeté, Governador Valadares, Ipatinga, Itambacuri, Itaobim, João Pinheiro, Patrocínio/Monte Carmelo, São Gotardo, Uberlândia/Araguari, and Unaí, when comparing the 2015–2019 and 2020–2023 periods. In the other micro-regions, the hidden prevalence of leprosy increased compared to the pre-pandemic period. As this is a composite indicator, this reduction is directly linked to the decrease in the number of new cases diagnosed, particularly those with disability (Table 3).

## 4. Discussion

The results of this study clearly demonstrate the impact of the COVID-19 pandemic on the epidemiological scenario of leprosy in Minas Gerais, especially in micro-regions classified as being at high risk of becoming ill. The drop in the detection rate of new cases intensified during the pandemic period, observed in several micro-regions, in line with the rates in Brazil. In addition to the reduction in overall detection, these rates also showed a reduction in detection among children under 15, compared to the period before the pandemic, contrasting with an increase in cases of multibacillary leprosy (MB) after the spread of COVID-19 in the country [11]. This was a global trend that has been identified in other endemic countries, such as India, where the pandemic resulted in a 63% reduction in new cases diagnosed in the second and third quarters of 2020 [6].

The barriers created by public health policies during the pandemic limited access to the health services needed to manage leprosy, impacting both clinical and community interventions [21]. Thus, this decrease in the detection rate is intrinsically linked to barriers to accessing health services [11] and the interruption of surveillance activities [22].

The hidden prevalence of leprosy, an indicator used to estimate the presence of undiagnosed cases, showed a drop in several micro-regions. This result indicates that there are fewer existing cases going undiagnosed in these locations. However, this reduction may not represent an advance in leprosy control, but rather the consequence of underreporting, especially with regard to disabled cases, during the pandemic period [23,24]. The proportion of cases reported without having the degree of disability assessed increased significantly during the pandemic [12]. The lack of assessment of disability among new cases and the consequent lack of registration of those possibly disabled interferes with the result of the hidden prevalence of leprosy, meaning that this indicator may not reflect the real situation of missed diagnoses.

In other micro-regions, there was an increase in the hidden prevalence of leprosy in the period following the most critical phase of the pandemic. This may be related to the greater detection of cases with a degree of disability already in place [25], emphasizing the vulnerability of these regions in leprosy control and pointing to the need for more incisive active search actions in order to reduce the hidden prevalence of leprosy and prevent the perpetuation of uncontrolled transmission chains [23].

With regard to the risk of falling ill, the data also show a heterogeneous impact of the pandemic in the different micro-regions of Minas Gerais. Some retained a high risk of falling ill, while in others the risk fell to medium, low, and even very low. These results should be interpreted with caution, considering the pandemic context in which delays in diagnosing new cases and underreporting have already been identified in the country [26]. The decrease in the number of diagnoses, resulting from underreporting, may mask the real severity of leprosy in these regions, and the perception of improvement may be illusory.

The reduction in notifications may not reflect a real drop in transmission, but rather a problem in the surveillance system, which has been severely impacted by the restrictions imposed by the pandemic [27]. Therefore, even in regions where there has been a reduction in the risk of illness, it is important to monitor this indicator, as well as others that portray the epidemiological scenario of leprosy as surveillance and diagnosis activities are resumed at full capacity.

The risk indicator is calculated from three epidemiological indicators of leprosy [20]. In this way, regions that have retained a high risk of falling ill should be monitored as areas of greater concern, especially those where there has been a drop in detection, as it indicates that the risk of falling ill is being impacted by the other two indicators, which represent detection in children under 15 and with grade 2 disability.

In these areas, the resumption of health services must be accompanied by an intensification of surveillance strategies, as well as training for professionals, so that control actions are carried out in a targeted manner, thus contributing to early diagnosis [28], and thereby avoiding the worsening of leprosy and its late complications.

There was no significant upturn in the detection of new leprosy cases in any of the micro-regions evaluated, nor in the state rate. However, Minas Gerais as a whole, and the micro-regions Governador Valadares, Ituiutaba, Uberaba, and Uberlândia/Araguari, showed a positive increase in this rate from 2021 onwards. This result suggests a resumption of leprosy surveillance actions.

When evaluating the initial resumption of detection in conjunction with the other indicators discussed in this study, it can be seen that in Governador Valadares and Uberlândia, the risk of becoming ill remained high and there was a reduction in the hidden prevalence of leprosy. These data can be seen as positive, since the risk is possibly being impacted by the increase in detection, which can be both general and in children under 15, which indicates active transmission of the disease. However, the reduction in the hidden prevalence of leprosy indicates that cases are being diagnosed without disabilities.

On the other hand, in Ituiutaba and Uberaba there was a resumption of detection with a reduction in the risk of becoming ill, but the hidden prevalence of leprosy increased. This result suggests that services need to pay more attention to resuming leprosy surveillance actions, since the estimate of the hidden prevalence of leprosy is strongly affected by the identification of disabled cases. In this way, it indicates the existence of trapped cases, as well as the need to strengthen health services and train professionals in the early identification of new cases of the disease [26,28].

The findings of this study show the complex interaction between the change in the detection rate and the indicators of the risk of illness and the hidden prevalence of leprosy. The numerous difficulties imposed by the pandemic period, such as difficulty in accessing health services [29], culminating in underdiagnosis and late diagnosis [26], have an even greater impact on the variations in these indicators.

The limitations of this study include the use of secondary SINAN data, which may contain inconsistencies or incompleteness due to the manual recording of data by the health professionals who enter the information into the system. Finally, the temporal interference of the pandemic in previous leprosy trends makes it difficult to separate the pandemic effects from the natural variations in the disease. It should be noted that the analysis of the time trend, carried out by calculating the annual percentage change (APC), could be more robust over a longer period of time.

## 5. Conclusions

With the data evaluated in this study, it was possible to observe the following: (a) a reduction in the detection of new cases in most of the micro-regions evaluated, considering the average of the pre-pandemic period in relation to the pandemic period; (b) an inflection point in the detection of new cases in the state rate and in some micro-regions, with a positive increase in the year 2021, making it possible to suggest a resumption of diagnoses; (c) a reduction in the hidden prevalence of leprosy observed in certain micro-regions, which is possibly linked to a reduction in diagnoses, especially in people with an disability; (d) an increase in the risk of falling ill with leprosy in the state of Minas Gerais; and (e) a reduction in the risk of leprosy disease in some micro-regions.

The pandemic has had a considerable impact on the detection of new cases in Minas Gerais and in the micro-regions evaluated. The heterogeneous results of both the risk of illness and the hidden prevalence of leprosy indicate that, even with the resumption of health services, there is still a long way to go to achieve the elimination of leprosy as a public health problem. It is imperative to implement public health policies that prioritize the early identification of cases, as well as ensuring that vulnerable populations are monitored.

The results of this study highlight the need for strategic post-pandemic interventions to mitigate the effects of interrupted services and ensure continuity of care. The results also reinforce the need for more robust surveillance and active search strategies, especially in historically underdiagnosed areas, to mitigate the negative impact caused by the health crisis and to reach the WHO 2030 global leprosy goal.

## Figures and Tables

**Table 1:** Overall detection rate of new cases per 100,000 inhabitants in Minas Gerais and health micro-regions, 2015 to 2023.

Micro-Region	2015	2016	2017	2018	2019	Average	2020	2021	2022	2023	Average
Almenara/Jacinto	20.53	22.85	17.55	25.11	26.24	22.46	12.81	18.03	9.89	10.47	12.80
Araçuaí	27.84	18.94	20.06	32.34	25.66	24.97	11.16	13.40	11.17	10.05	11.44
Belo Horizonte/Nova Lima/Caeté	3.32	3.57	3.94	2.86	2.90	3.32	1.58	2.03	2.12	1.74	1.87
Carangola	4.70	3.91	2.34	2.34	3.11	3.28	3.10	1.55	0.77	2.32	1.94
Governador Valadares	19.76	24.62	18.70	19.57	22.99	21.13	12.50	13.85	17.31	19.16	15.71
Ipatinga	9.58	7.56	6.30	5.77	4.77	6.80	4.27	4.71	5.42	3.53	4.48
Itambacuri	35.84	24.66	26.91	78.56	56.15	44.42	20.23	47.23	29.24	49.48	36.54
Itaobim	12.28	35.68	16.02	12.35	19.80	19.23	16.11	4.97	6.21	4.97	8.06
Ituiutaba	13.02	9.33	9.81	12.33	7.68	10.43	9.69	5.59	5.59	8.63	7.37
Janaúba/Monte Azul	3.27	2.54	2.53	2.88	4.67	3.18	2.15	3.57	2.50	2.86	2.77
João Pinheiro	16.86	16.72	4.15	2.74	5.44	9.18	1.35	16.08	1.34	2.68	5.36
Patos de Minas	8.87	6.75	4.65	8.23	6.66	7.03	2.55	5.07	5.07	3.55	4.06
Patrocínio/Monte Carmelo	1.95	1.93	3.07	3.05	1.14	2.23	1.51	0.38	0.75	0.38	0.75
Ponte Nova	2.34	1.88	4.24	4.72	4.73	3.58	0.95	0.95	1.43	1.90	1.31
Resplendor	33.55	32.45	57.08	16.80	31.37	34.25	8.97	15.70	2.24	11.21	9.53
São Gotardo	3.21	1.07	3.19	7.43	4.23	3.83	4.22	7.36	6.31	7.36	6.31
Uberaba	4.49	7.15	4.14	6.02	3.81	5.12	2.12	1.64	2.34	2.80	2.22
Uberlândia/Araguari	14.37	17.25	17.07	14.46	11.25	14.88	5.52	6.11	13.82	8.79	8.56
Unaí	16.86	11.91	13.30	16.15	15.31	14.71	10.14	9.00	9.36	9.36	9.46
Minas Gerais	5.51	5.41	5.32	4.95	5.22	5.28	3.33	4.05	4.92	5.27	4.39

**Table 2:** Leprosy Disease Risk Index in Minas Gerais and health micro-regions, 2015 to 2023.

Micro-Regional	2015–2019		2020–2023	
Almenara/Jacinto	0.49	High	0.51	High
Araçuaí	0.54	High	0.51	High
Belo horizonte/Nova Lima/Caeté	0.47	High	0.70	High
Carangola	0.44	High	0.46	Medium
Governador Valadares	0.57	High	0.78	High
Ipatinga	0.50	High	0.58	High
Itambacuri	0.55	High	0.58	High
Itaobim	0.44	High	0.42	Medium
Ituiutaba	0.55	High	0.42	Medium
Janaúba/Monte Azul	0.44	High	0.51	High
João Pinheiro	0.46	High	0.11	Very Low
Patos de Minas	0.47	High	0.43	Medium
Patrocínio/Monte Carmelo	0.54	High	0.17	Very Low
Ponte Nova	0.50	High	0.31	Low
Resplendor	0.48	High	0.40	Medium
São Gotardo	0.44	High	0.62	High
Uberaba	0.51	High	0.35	Low
Uberlândia/Araguari	0.74	High	0.54	High
Unaí	0.53	High	0.73	High
Minas Gerais	0.28	Medium	0.55	High

**Table 3:** Hidden prevalence of leprosy cases in Minas Gerais and health micro-regions, 2015 to 2023.

Micro-Region	Average 2015–2019			Average 2020–2023		
	Hidden	Expected	Actual	Hidden	Expected	Actual
Almenara/Jacinto	2.25	89.93	92.18	2.32	32.34	34.66
Araçuaí	1.52	35.38	36.90	2.41	19.60	22.01
Belo horizonte/Nova Lima/Caeté	2.67	343.62	346.28	2.37	83.86	86.23
Carangola	-	-	-	3.60	6.84	10.45
Governador Valadares	3.77	412.77	416.54	2.33	71.61	73.94
Ipatinga	2.95	86.90	89.86	2.76	22.39	25.15
Itambacuri	3.77	51.02	54.79	2.85	19.51	22.36
Itaobim	3.06	38.59	41.65	2.03	14.09	16.11
Ituiutaba	2.60	73.61	76.21	2.90	18.84	21.74
Janaúba/Monte Azul	2.39	21.07	23.46	2.54	9.61	12.15
João Pinheiro	2.55	38.74	41.28	1.17	4.87	6.04
Patos de Minas	0.96	14.44	15.40	3.05	13.38	16.43
Patrocínio/Monte Carmelo	4.04	33.18	37.22	3.21	8.01	11.22
Ponte Nova	2.64	17.73	20.37	2.83	9.11	11.93
Resplendor	2.75	66.21	68.96	3.29	25.03	28.32
São Gotardo	3.36	13.83	17.18	1.81	5.36	7.16
Uberaba	3.09	94.12	97.21	3.13	19.26	22.39
Uberlândia/Araguari	3.27	366.03	369.30	2.33	95.70	98.03
Unaí	2.81	131.14	133.95	1.84	32.61	34.46
Minas Gerais	2.78	1243.82	1246.61	2.00	1013.15	1015.16

## Data Availability

Data can be made available by the authors upon request.

## References

[1] Alter A., Grant A., Abel L., Alcaïs A., Schurr E. (2011). Leprosy as a genetic disease. Mamm. Genome.

[2] Nath I., Saini C., Valluri V.L. (2015). Immunology of leprosy and diagnostic challenges. Clin. Dermatol..

[3] Leano H.A.d.M., Araújo K.M.d.F.A., Bueno I.d.C., Niitsuma E.N.A., Lana F.C.F. (2019). Fatores socioeconômicos relacionados à hanseníase: Revisão integrativa da literatura. Rev. Bras. Enferm..

[4] Nery J.S., Ramond A., Pescarini J.M., Alves A., Strina A., Ichihara M.Y., Penna M.L.F., Smeeth L., Rodrigues L.C., Barreto M.L. (2019). Socioeconomic determinants of leprosy new case detection in the 100 Million Brazilian Cohort: A population-based linkage study. Lancet Glob. Health.

[5] World Health Organization (2023). Weekly Epidemiological Record.

[6] De Arquer G.R., Kumar A., Singh R.K., Satle N., Mamidi R., Biswas P. (2021). COVID-19 and leprosy new case detection in India. Lepr. Rev..

[7] Vieira N.F., Martínez-Riera J.R., Lana F.C.F. (2020). Primary care quality and its effects on leprosy monitoring indicators. Rev. Bras. Enferm..

[8] Dantas R.C.C., De Campos P.A., Rossi I., Ribas R.M. (2022). Implications of social distancing in Brazil in the COVID-19 pandemic. Infect. Control Hosp. Epidemiol..

[9] Brasil (2020). Conselho Nacional de Saúde—RECOMENDAÇÃO No 030, DE 27 DE ABRIL DE 2020. Recomenda Medidas Que Visam a Garantia Dos Direitos e da Proteção Social das Pessoas Com Doenças Crônicas e Patologias [Internet]. http://conselho.saude.gov.br/recomendacoes-cns/1143-recomendacao-n-030-de-27-de-abril-de-2020.

[10] Minas Gerais (2020). Eduardo de Menezes é o Primeiro Hospital Mineiro Integralmente Destinado ao Atendimento de Pacientes COVID-19|Secretaria de Estado de Saúde de Minas Gerais [Internet]. https://www.saude.mg.gov.br/component/gmg/story/12436-eduardo-de-menezes-e-o-primeiro-hospital-mineiro-integralmente-destinado-ao-atendimento-de-pacientes-covid-19.

[11] Paz W.S., Souza M.R., Tavares D.S., de Jesus A.R., Santos A.D., Carmo R.F., de Souza C.D.F., Bezerra-Santos M. (2022). Impact of the COVID-19 pandemic on the diagnosis of leprosy in Brazil: An ecological and population-based study. Lancet Reg. Health.

[12] Matos T.S., do Nascimento V.A., do Carmo R.F., Moreno de Oliveira Fernandes T.R., de Souza C.D.F., da Silva T.F.A. (2021). Impact of the COVID-19 pandemic on the diagnosis of new leprosy cases in Northeastern Brazil, 2020. Int. J. Dermatol..

[13] Organização Mundial da Saúde (2021). Estratégia Global de Hanseníase 2021–2030—“Towards Zero Leprosy” [Internet]. https://www.who.int/pt/publications/i/item/9789290228509.

[14] Brasil (2024). Estratégia Nacional Para Enfrentamento à Hanseníase 2024–2030.

[15] Minas Gerais (2019). Plano de Enfrentamento da Hanseníase em Minas Gerais, 2019–2022.

[16] Bueno I.d.C., Lages D.d.S., Lana F.C.F. (2023). Spatial analysis of the epidemiological risk of leprosy in the municipalities of Minas Gerais. PLoS Negl. Trop. Dis..

[17] Ignotti E., Rodrigues A.M., Andrade VLG de Valente J.G. (2004). Aplicação de métodos de estimativa da prevalência de hanseníase no Estado de Mato Grosso. Rev. Bras. Epidemiol..

[18] Brasil (2016). Diretrizes Para Vigilância, Atenção e Eliminação da Hanseníase Como Problema de Saúde Pública: Manual Técnico-Operacional.

[19] Lombardi C., Suárez R., Talhari S., Neves R.G. (1997). Epidemiologia da Hanseníase.

[20] Araújo K.M.d.F.A., Gomes L.C.F., Lana F.C.F. (2020). Análise espacial do risco de adoecimento da hanseníase em um estado do nordeste Brasileiro. Rev. Baiana Enferm..

[21] Mahato S., Bhattarai S., Singh R. (2020). Inequities towards leprosy-affected people: A challenge during COVID-19 pandemic. PLoS Negl. Trop. Dis..

[22] Thangaraju P., Arulmani M., Venkatesan S., Gurunthalingam M., Thangaraju E. (2020). COVID-19 and leprosy-hurdles and possible solutions. Asian Pac. J. Trop. Med..

[23] da Cunha V.P., Botelho G.M., de Oliveira A.H.M., Monteiro L.D., de Barros Franco D.G., da Costa Silva R. (2021). Application of the ARIMA Model to Predict Under-Reporting of New Cases of Hansen’s Disease during the COVID-19 Pandemic in a Municipality of the Amazon Region. Int. J. Environ. Res. Public Health.

[24] Dominic S., Sasidharanpillai S., Gangan R., Minu U., Sneha K.S., Hameed J., Devi K. (2022). Impact of Lockdown Restrictions on Treatment of Leprosy. Indian. Dermatol. Online J..

[25] de Souza C.D.F., Santos F.G.B. (2019). Prevalência da hanseníase, taxa de grau II de incapacidade física e proporção de casos multibacilares: Um paradoxo que evidencia diagnóstico tardio e prevalência oculta?. Rev. De Epidemiol. E Controle Infecção.

[26] Ziembowicz H., Souza I., Peruzzo J.V., Subtil L.d.C., Onófrio L.G., Vaucher M.B. (2022). As consequências da pandemia de Sars-CoV-2 sobre a educação médica no combate à hanseníase. Rev. Epidemiol. E Controle Infecção.

[27] Pschichholz L. (2022). Impacto da pandemia de SARS-CoV-2 na incidência de hanseníase no brasil: Comparação com os últimos 5 anos. Braz. J. Infect. Dis..

[28] Vieira N.F., Lanza F.M., Martínez-Riera J.R., Nolasco A., Lana F.C.F. (2020). Orientación de la atención primaria en las acciones contra la lepra: Factores relacionados con los profesionales. Gac. Sanit..

[29] de Barros B., Lambert S.M., Negera E., de Arquer G.R., Sales A.M., Darlong J., Dias V.L.A., Rozario B.J., Pai V.V., Alinda M.D. (2021). An assessment of the reported impact of the COVID-19 pandemic on leprosy services using an online survey of practitioners in leprosy referral centres. Trans. R. Soc. Trop. Med. Hyg..

